# Effective Restoration of Gastric and Esophageal Tissues in an In Vitro Model of GERD: Mucoadhesive and Protective Properties of Xyloglucan, Pea Proteins, and Polyacrylic Acid

**DOI:** 10.3390/ijms26094409

**Published:** 2025-05-06

**Authors:** Sara Ferrari, Federica Ferulli, Rebecca Galla, Riccardo Vicini, Veronica Cattaneo, Simone Mulè, Francesca Uberti

**Affiliations:** 1 Laboratory of Physiology, Department for Sustainable Development and Ecological Transition, University of Piemonte Orientale, UPO, 13100 Vercelli, Italy; 2 Bio Basic Europe Srl, 20146 Milan, Italy; 3 Noivita Srls, Spin Off, Department for Sustainable Development and Ecological Transition, University of Piemonte Orientale, Via Solaroli 17, 28100 Novara, Italy

**Keywords:** GERD, xyloglucan, pea proteins, permeability, mucoadhesion, barrier effect

## Abstract

Esophageal barrier dysfunction is a crucial pathophysiological mechanism of gastroesophageal reflux disease (GERD). However, treatments mainly aim to reduce gastric acidity rather than improve tissue integrity. This study evaluated the protective and mucoadhesive properties of a formulation containing xyloglucan, pea proteins, and polyacrylic acid (XPPA) in gastric and esophageal cells. Cells were exposed to hydrochloric acid (HCl) and subsequently treated with the test compound. Trans-epithelial electrical resistance (TEER), tight junction (TJ) expression, and mucoadhesion of XPPA on gastric and esophageal cells were evaluated. To further confirm the protective ability of XPPA, a Lucifer Yellow assay was performed on a human reconstructed esophageal epithelium to assess the ability of XPPA to prevent HCl-induced hyperpermeability. XPPA possesses noteworthy mucoadhesive properties, ensuring an extended contact time between the product and the damaged mucosa to allow sustained mucosal protection. Furthermore, XPPA effectively restored gastroesophageal barrier integrity after HCl-induced damage, as assessed with TEER, after 1 h (*p* < 0.05). Finally, XPPA helped to restore TJ expression (*p* < 0.05) and protected the tissues from hyperpermeability for at least 2 h (*p* < 0.05). These results pave the way for using XPPA as a promising treatment to ameliorate gastroesophageal barrier properties in GERD patients.

## 1. Introduction

Gastroesophageal reflux disease (GERD) is a worldwide prevalent pathological condition, caused by abnormal reflux of stomach contents into the esophagus [1], with an incidence of up to 20% in Western countries [2]. Heartburn and regurgitation represent the two cardinal symptoms of GERD. However, the symptomatology includes a wide range of extraesophageal manifestations that can vary from patient to patient, such as laryngopharyngeal inflammation causing cough, swallowing dysfunction, and chest and epigastric pain [3,4,5]. The etiology of this disease is complex and multifactorial, including an imbalance between several factors, including alterations of mucosal defensive factors [6,7].

Mucosal barrier integrity along the gastroesophageal tract is crucial to sustain tissue function and to prevent the translocation of noxious substances from the lumen to the submucosa. Among the structural elements maintaining the integrity of the tissue, tight junctions (TJs) play a significant role in holding the cells adjacent, providing the epithelium with its barrier properties [8,9].

Esophageal tissue possesses effective barrier properties characterized by intrinsic defensive mechanisms that protect the cells from physiological luminal acidity [10,11]. However, it has been demonstrated that GERD patients cannot maintain a functional esophageal epithelial barrier. Indeed, an abnormal repeated exposure of the esophagus to gastric secretions containing irritating agents such as hydrochloric acid (HCl) and pepsins might cause an imbalance between the defensive mechanisms and the aggressive insults of gastric refluxate, leading to increased paracellular permeability, dilated intracellular spaces, and structural alterations in the extracellular matrix (ECM) at the esophageal level [10,12]. Notably, an acid-dependent reduction in expression of TJ proteins claudin-1 and claudin-4 in adhesion to ECM components such as vitronectin and fibronectin in esophageal tissues has been observed in GERD patients and models [12,13,14].

This increased esophageal permeability facilitates the contact between the gastric refluxate, mucosal esophageal chemoreceptors, and sensory nerve endings in the chest region, resulting in the perception of heartburn [15,16,17,18].

Although GERD is linked to esophageal epithelial dysfunction, most first-line therapies focus on reducing gastric hyperacidity, with little or no regard for preserving tissue integrity [19]. Therefore, targeting preserving and restoring the esophageal epithelial barrier while controlling gastric acidity may represent an important therapeutic strategy to improve symptoms and prolong remission in GERD patients [20].

To date, the cornerstone of GERD treatment is proton pump inhibitors (PPIs), in conjunction with suitable lifestyle changes for individual patients. PPIs have a relatively good safety record; nonetheless, they have some limitations: they can only inhibit proton pump molecules (H^+^/K^+^-ATPase), have a short pharmacokinetic half-life, are acidic, have a slow onset of action, and have varied pharmacodynamic effects because of many genetically determined polymorphisms of cytochrome P450 2C19 [21]. Unlike conventional PPIs, a new possible treatment called vonoprazan binds to the potassium ion binding site and competitively inhibits H^+^, K^+^-ATPase without requiring stomach acid activation. In some countries, vonoprazan has been approved to aid in healing reflux esophagitis and avoid recurrence. However, currently available studies have several limitations, such as small sample sizes, the absence of evaluation of endoscopic or histological parameters, which could provide a more accurate assessment of GERD, and substantial heterogeneity in most outcomes [22].

In this context, naturally derived compounds are currently being investigated as promising treatments for several gastrointestinal tract diseases, including GERD. For instance, *Aloe barbadensis* and brown seaweed polysaccharides were evaluated for their barrier properties and antioxidant activities in vitro, where the compounds prevented the cellular damage caused by an acidic insult [23]. However, clinical evidence in this regard is limited. Therefore, there is a need for more in-depth studies addressing the efficacy of those ingredients in patients [24,25].

Recently, particular interest has been given to non-pharmacological compounds with mucoadhesive and mucoprotective properties, such as specific polysaccharides and proteins.

Xyloglucan (XG) is a polysaccharide extracted from the seeds of the tamarind tree that is widely used as a thickening and stabilizing agent in various food and medicinal products [26]. The mucoadhesive and mucoprotective properties of XG are conferred by its chemical structure, which resembles that of the gastrointestinal protein “mucin” [27]. Indeed, XG can form a protective layer over the human intestinal mucosa, restoring intestinal epithelial cell function [28,29,30]. Several in vitro models of intestinal diseases have shown that XG favors TJ integrity and helps restore mucosal permeability of gastrointestinal epithelia [31,32]. Furthermore, it has been proven that the mechanical barrier formed by XG prevents epithelial cells from contacting pathogens and their byproducts, thus acting against bacterial adhesion and invasion [29,32,33,34].

Pea protein (PP), derived from high-quality *Pisum sativum*, is a highly digestible source of amino acids and fibers. It also has gelling properties and an optimal safety profile [35].

XG and PP have previously worked synergistically to restore intestinal epithelial cells’ barrier properties. Specifically, the combination of XG and PP was able to preserve the integrity of TJ proteins Zonula Occludens-1 (ZO-1) and occludin, maintaining the barrier integrity of intestinal mucosae in different preclinical models of gastrointestinal symptoms [36,37].

Polyacrylic acid (PA) is a well-known mucoadhesive polymer that has been extensively studied for its potential in mucosal substance delivery applications, as it has been demonstrated to prolong the residence time of various formulations on mucosal surfaces such as the gastrointestinal tract, thereby enhancing their efficacy [38].

Interestingly, the combination of XG, PP, and PA (hereinafter referred to as “XPPA”) was able to restore gastric and esophageal homeostasis in a murine model of GERD by reducing gastric and esophageal damage after 7 days, as well as epigastric pain and cytokine levels after 3 days of treatment [39].

This study investigated the mechanism of action of XPPA through different in vitro models. Specifically, it aimed to assess the ability of XPPA to form a mucoadhesive barrier on gastroesophageal epithelia, protect the tissues from external insults, and promote gastroesophageal barrier integrity.

## 2. Results

### 2.1. Cell Viability and ROS Production of Gastric Epithelial Cells

**Figure 1:**
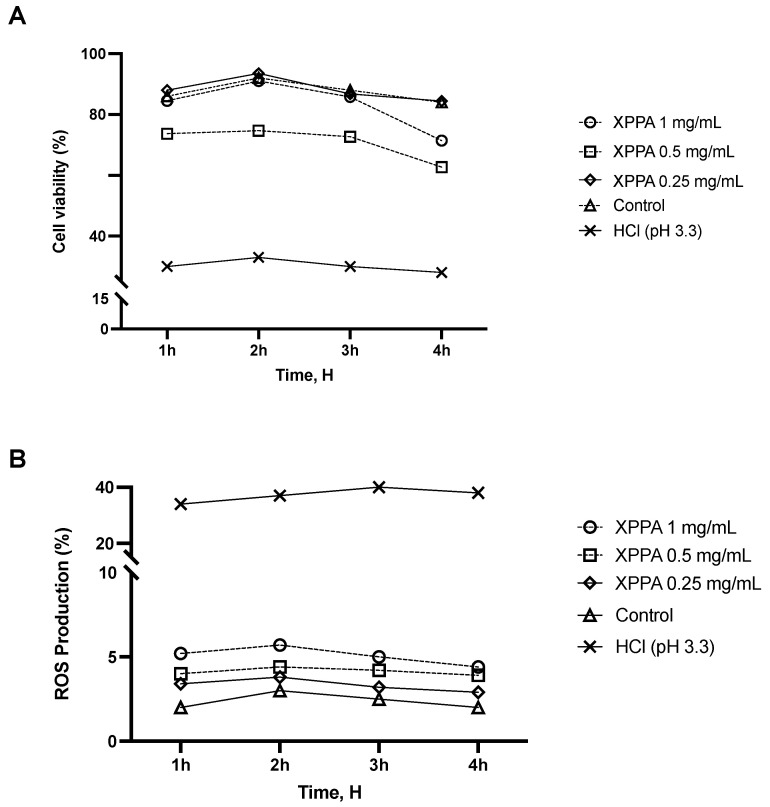
Effects of XPPA on GTL-16 cell viability and ROS production under hyperacidity conditions. (**A**) Cell viability was measured by the MTT test; (**B**) ROS production analysis was measured through cytochrome C reduction. HCl = positive control, cells in GERD condition induced by HCl; Control = negative control, untreated cells. Data are expressed as means ± SD (%) of 5 independent experiments. All the concentrations of XPPA significantly improved both cell viability and ROS production, compared with positive controls (*p* < 0.05 for all concentrations, at all time points). HCl: hydrochloric acid; MTT: 3-(4,5-Dimethylthiazol-2-yl)-2,5-diphenyltetrazolium bromide; ROS: reactive oxygen species; XXPA: combination of xyloglucan, pea protein, and polyacrylic acid.

In the first set of experiments, a time-course study was performed to determine the effect of XPPA on cell viability in a 3D model of gastric tissue. GTL-16 cells were treated with HCl (pH 3.3) to mimic hyperacidity conditions characteristic of GERD and were then treated with XPPA at different concentrations. Cell viability was assessed over 4 h (at 1 h, 2 h, 3 h, and 4 h). Figure 1A shows that positive control cells showed a significantly lower viability than negative controls (*p* < 0.05). Interestingly, all XPPA concentrations could counteract the effect induced by HCl (*p* < 0.05) at all time points analyzed.

Reactive oxygen species (ROS) are associated with epithelial damage in reflux esophagitis and GERD [40]. An additional time-dependent study was conducted using the same in vitro model to assess the potential impact of XPPA on ROS production. The detrimental effects of HCl on ROS production observed in positive controls (*p* < 0.05) were mitigated by all concentrations of XPPA (Figure 1B). Indeed, a statistically significant difference in ROS production was observed between positive controls and XPPA-treated cells (*p* < 0.05 for all concentrations, at all time points. Concerning the concentrations tested, it would appear that there is a dose-dependent effect inversely proportional to concentration; indeed, XPPA 1 mg/mL, although still inducing beneficial effects compared to the harm condition (*p* < 0.05), induces a smaller effect than either of the other two lower concentrations in both parameters analyzed. In line with this, XPPA 0.5 mg/mL induces better effects than the 1 mg/mL concentration, but still slightly lower than XPPA 0.25 mg/mL.

### 2.2. Effects of XPPA on Gastric Barrier Integrity

**Figure 2:**
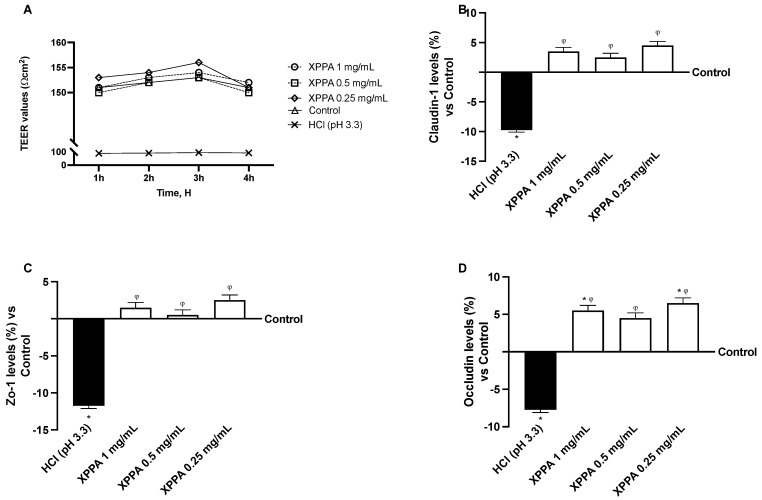
Evaluation of barrier integrity of GTL-16 cells. (**A**) TEER (transepithelial electrical resistance) value measured using EVOM3 at different timepoints (from 1 h to 4 h); (**B**–**D**) analysis of TJ measured by Enzyme-Linked Immunosorbent Assay (ELISA) test (Claudin-1, ZO-1, and occludin, respectively) at 4 h. HCl = positive control, cells in GERD condition induced by HCl; Control = negative control, untreated cells. Data are expressed as means ± SD (%) of 5 independent experiments. * *p* < 0.05 vs. negative control; φ *p* < 0.05 vs. positive control. HCl: hydrochloric acid; TEER: trans-epithelial electrical resistance; XXPA: combination of xyloglucan, pea protein, and polyacrylic acid; ZO-1: Zonula Occludens-1.

Trans-epithelial electrical resistance (TEER) was measured in GTL-16 cells to assess barrier integrity. HCl significantly reduced TEER (*p* < 0.05), demonstrating the harmful effect of hyperacidity on gastric cells. Notably, all XPPA concentrations were able to significantly increase TEER values (*p* < 0.05 for all concentrations, at all time points), thus restoring barrier integrity to a physiological level (Figure 2A).

The expression of TJ proteins was analyzed to support this result further. Claudin-1, occludin, and ZO-1 levels showed a strong decrease upon HCl treatment, compared to untreated cells (*p* < 0.05). In contrast, all XPPA concentrations restored the physiological expression of all three junction proteins (Figure 2B–D).

### 2.3. Influence of XPPA on Vitronectin and Fibronectin Quantification

**Figure 3:**
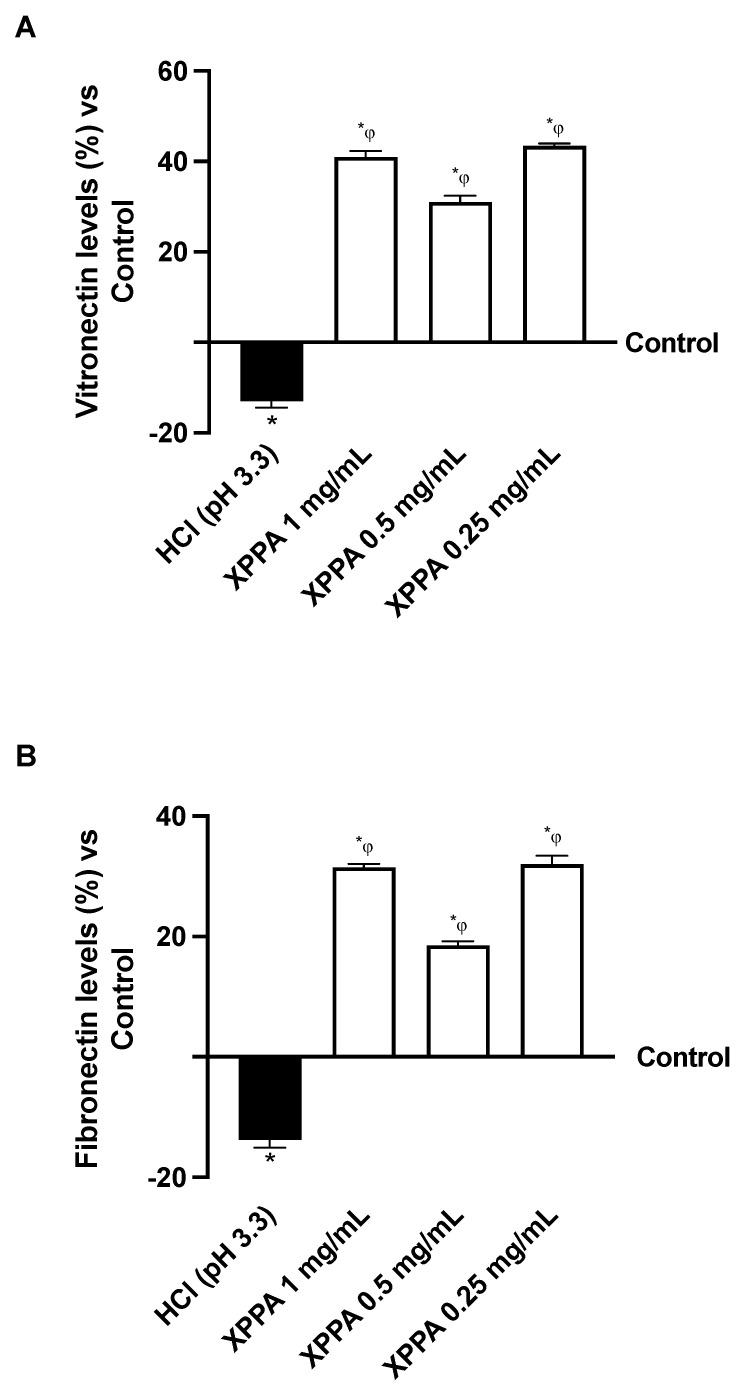
Adhesion study on GTL-16 cells. (**A**) Vitronectin and (**B**) fibronectin analysis measured by Enzyme-Linked Immunosorbent Assay (ELISA) test at 4 h. HCl = positive control, cells in GERD condition induced by HCl; Control = negative control, untreated cells. Data are expressed as means ± SD (%) of 5 independent experiments. * *p* < 0.05 vs. negative control; φ *p* < 0.05 vs. positive control. HCl: hydrochloric acid; XXPA: combination of xyloglucan, pea protein, and polyacrylic acid.

The effects of XPPA on cellular adhesion capacity in hyperacidic conditions were evaluated. Specifically, two extracellular matrix glycoproteins known to be involved in adhesion, vitronectin and fibronectin, were quantified using ELISA to examine the effects of gastric acid on cell adhesion to the extracellular matrix (Figure 3). As expected, HCl determined a decrease in the expression of vitronectin and fibronectin, indicating a loss of adhesion compared to the negative control (*p* < 0.05). Notably, XPPA re-established the expression levels of both glycoproteins, possibly indicating the restoration of cellular adhesion to the ECM (*p* < 0.05 vs. both positive and negative controls).

### 2.4. Mucoadhesive Potential of XPPA

**Figure 4:**
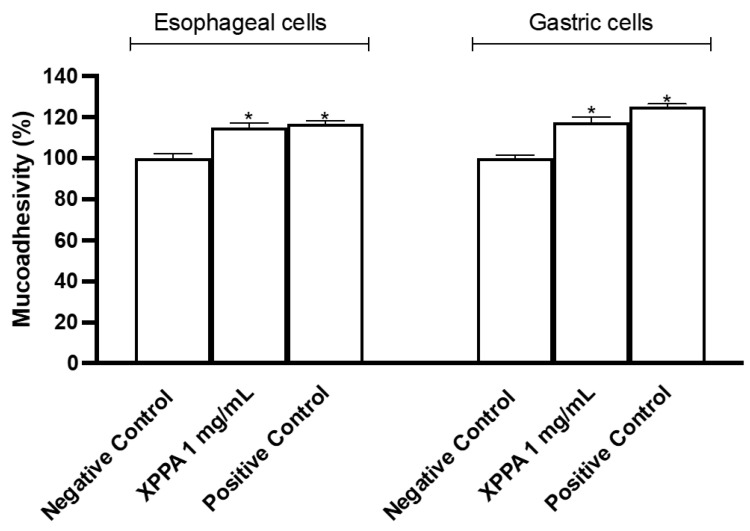
Mucoadhesion test on esophageal cells (KYSE-30) and on gastric cells (AGS). The values are expressed as means ± SD. Negative control = untreated cells, positive control = substance with known mucoadhesive properties. * *p* < 0.05 vs. negative control. XXPA: combination of xyloglucan, pea protein, and polyacrylic acid.

Mucoadhesion is a crucial feature of GERD treatments, as it allows the formulation to stay in contact with gastroesophageal tissues for a prolonged time. The mucoadhesive property of XPPA was tested both in gastric and esophageal cells (Figure 4). The concentration evaluated was chosen based on the cell viability test. Indeed, the tested product showed significant mucoadhesive properties on both esophageal and gastric cells, compared with the negative control (*p* < 0.05).

### 2.5. XPPA Effects on Mucosal Permeability

**Figure 5:**
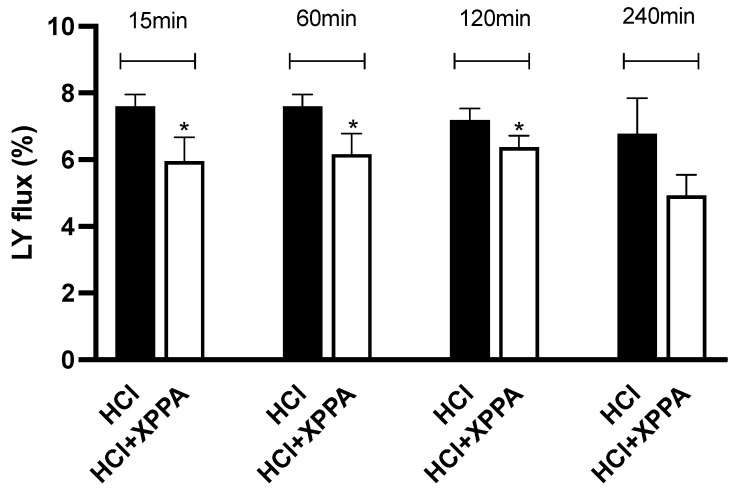
Percentage of Lucifer Yellow flux after 15, 60, 120, and 240 min of stimulation with HCl in the presence and absence of the tested product. Triplicate human reconstructed esophageal epithelium (HO2E) tissues were used for each treatment. The values are expressed as means ± SD. * *p* < 0.05 vs. HCl (positive control). HCl: hydrochloric acid; LY: luciferase yellow; XXPA: combination of xyloglucan, pea protein, and polyacrylic acid.

To further evaluate the protective effects of XPPA against damages induced by GERD, an in vitro model of HO2E was used (Figure 5). The tissue was pre-treated with XPPA, and HCl was then used to simulate GERD-like damage. Lucifer Yellow (LY), a fluorescent probe, was used to measure paracellular flux. Results show that LY flux was significantly lower in tissues treated with XPPA after 15, 60, and 120 min of stimulation with HCl, compared to untreated cells (*p* < 0.05), demonstrating that the product was able to maintain the integrity of human esophageal epithelium under hyperacidity conditions.

## 3. Discussion

A dysfunctional esophageal epithelial barrier has been observed in GERD patients, who display a reduced TJ expression and subsequent increased esophageal permeability [10]. Although GERD is a common medical problem, it can be difficult to diagnose and treat, especially if the symptoms are unusual, and can significantly affect the quality of life. As for conventional treatments, PPIs are safe and well tolerated; however, they may be associated with side effects such as community-acquired pneumonia, *Clostridium difficile* infection, and chronic kidney disease. Therefore, new treatments must be identified for optimal management of GERD and to minimize side effects [41]. In addition, long-term use of PPIs can cause a number of side effects, such as vitamin B12, magnesium, and calcium deficiency, which increases the risk of osteoporosis and bone fractures [42].

For this reason, natural compounds with mucoprotective and non-pharmacological actions have recently gained attention as potential therapeutic agents able to restore the integrity of the esophageal mucosa, possibly lengthening remission periods and postponing relapses [20]. In light of this, the focus of the present study was to investigate the mechanism of action of XPPA, a novel product intended for oral intake containing xyloglucan, pea protein, and polyacrylic acid, in different in vitro GERD models, as these ingredients are known for their promising mucoprotective and mucoadhesive properties [27,38].

In this study, exposing gastroesophageal cells to HCl caused GERD-like damage. As expected, this caused a significant decrease in cell vitality, while post-treatment with XPPA significantly restored viability, highlighting the compound’s ability to protect the cells from acid-induced damage. The restoration of cell viability is particularly important considering that it was demonstrated that pepsin, a component of gastric refluxate, can cause epithelial damage and death through different mechanisms [43].

The development of GERD, similarly to other common human gastrointestinal diseases, is associated with tissue inflammation and increased oxidative stress [44]. Indeed, cellular exposure to acid damage is known to cause increased production of ROS [45]. Therefore, ROS production following HCl exposure and treatment with XPPA was analyzed, revealing that XPPA-treated cells showed a significantly lower level of ROS production. This outcome might be explained through an indirect effect of XPPA, which allows the natural tissue healing and restoration of cellular homeostasis with its mechanical protective properties.

Continuous exposure to the aggressive gastric refluxate is known to cause structural damage in cell junctions and ECM components, thus compromising esophageal barrier integrity [10,12]. This correlates with an increased permeability across the epithelial esophageal tissue, which may allow H^+^ protons from acid refluxate to come in contact with sensory nerve endings, causing painful symptoms [17]. In this context, gastric barrier integrity was analyzed through TEER and TJ quantification to unravel the ability of XPPA to restore epithelial integrity under hyperacidity conditions. Moreover, the expression of fibronectin and vitronectin was investigated to assess the cellular adhesion capacity. Indeed, acidic exposure caused a loss of barrier function. At the same time, treatment with XPPA increased TEER levels and restored claudin-1, ZO-1, and occludin levels while promoting vitronectin and fibronectin expression, proving its positive effect on tissue integrity. These results are noteworthy, as it has been hypothesized that acid-induced down-regulation of TJ’s proteins may help to explain the esophageal barrier dysfunction observed in GERD patients. However, further studies are needed to define this alteration’s mechanisms and functional consequences. The ability of XPPA to promote esophageal barrier integrity was further corroborated by the outcome of a permeability assay with LY, performed on human reconstructed esophageal epithelium. Interestingly, these results correlate with the positive outcome of a previous study in which XPPA was able to reduce both macroscopical and microscopical gastric and esophageal damage, as well as epigastric pain and cytokine levels, in a murine model of GERD [39]. This suggests that restoring epithelial barrier integrity might be the primary mechanism of action by which XPPA can promote gastroesophageal homeostasis, counteracting signs and symptoms of GERD.

Mucoadhesion may be defined as the state in which interfacial forces hold a substance and a mucous membrane together for extended periods [46]. In the last decades, mucoadhesion has received notable interest for its potential in optimizing drug delivery, as it allows a substance to increase the contact time at the sites of application on the mucosae, prolonging the residence time and therefore prolonging its beneficial effects. Furthermore, mucoadhesive materials might possess intrinsic therapeutic properties, especially in the mechanical protection of damaged tissues [47]. In this context, XPPA displayed significant mucoadhesive properties on both gastric and esophageal cells. This would ensure an extended contact time between the product and the damaged gastroesophageal mucosa, allowing sustained mucosal protection.

In vitro three-dimensional tissue models represent a promising alternative to replace animal testing for preclinical evaluation of the efficacy and safety of products. Indeed, in respect to cell monolayers, 3D models are more complex and able to mimic in vivo human tissues in terms of morphological, biochemical, and physiological aspects [48,49]. Three-dimensional in vitro models are considered among the most reliable approaches to investigate the potential effects of compounds whose main mechanism of action consists of interaction with epithelial tissues, such as mucoprotectants [50].

In light of the successful outcome of this study, further preclinical assessments might be necessary for a deeper understanding of the mechanical activity of the product—for example, to explore the duration of XPPA’s mechanical barrier in both gastric and esophageal tissues and to evaluate the bioadhesive capacity of the product after oral intake.

Although in vitro models provide a realistic micro-environment for studying tissue responses, they may not fully represent the physiological status within the human stomach and esophagus. They cannot consider important aspects of the gastroesophageal tissue complexity, such as the interplay between epithelial tissues and mucus, lymphatic and blood vessels, and the entirety of the refluxate components.

For this reason, clinical investigations will be essential to confirm the importance of XPPA’s protective action and safety in pathological conditions involving abnormal reflux, such as GERD. Thanks to its promising beneficial effect and the safe nature of the singular ingredients, XPPA might become a promising ally in the long-term control of chronic or recurrent symptoms, such as GERD.

## 4. Materials and Methods

### 4.1. Agent Preparation

XPPA was prepared with the following functional ingredients: 13.5 mg/mL of xyloglucan (tamarind seed polysaccharide, MP Gokyo Food & Chemical Co., Ltd., Osaka, Japan), 24 mg/mL pea protein (proteins from *Pisum sativum*, COSUCRA Groupe Warcoing S.A, Warcoing, Belgium), and 2.5 mg/mL polyacrylic acid (Carbopol polymer, the Lubrizol Corporation, Wickliffe, OH, USA).

### 4.2. Cell Culture

GTL-16 is a clonal cell line derived from a poorly differentiated gastric carcinoma cell line, purchased by American Type Culture Collection (ATCC, Manassas, VA, USA) [51]. Cells were cultured in Dulbecco’s Modified Eagle Medium (DMEM, Merck Life Science, Rome, Italy) supplemented with 10% fetal bovine serum (FBS, Merck Life Science, Rome, Italy) and 1% penicillin–streptomycin (Merck Life Science, Rome, Italy) and incubated at 37 °C with 5% CO_2_ [52]. Different plating methods were used for the cells, including 1 × 10^4^ cells on 96-well plates to study cell viability and ROS production after synchronizing cells for 8 h with DMEM without red phenol, supplemented with 0.5% FBS (Merck Life Science, Rome, Italy), 2 mM L-glutamine, and 1% penicillin–streptomycin at 37 °C, and 5 × 10^5^ cells on 6.5 mm Transwell^®^ (Corning^®^ Costar^®^, Merck Life Science, Rome, Italy) with a 0.4 μm pore polycarbonate membrane insert (Corning^®^ Costar^®^, Merck Life Science, Rome, Italy) in a 24-well plate to perform the integrity and adhesion analyses [53].

Mucoadhesion tests were performed on both a gastric cell line (AGS) and an esophageal cell line (KYSE-30).

AGS is a cell line exhibiting epithelial morphology, isolated from gastric adenocarcinoma purchased by ATCC (Manassas, VA, USA) [54]. The cells were maintained in Ham’s F-12 supplemented with 10% FBS (Merck Life Science, Rome, Italy) and 1% penicillin–streptomycin and incubated at standard culture conditions (37 °C and 5% CO_2_). To perform mucoadhesion tests, 2.0 × 10^4^ cells were plated on 96-well plates [55].

Human KYSE-30 esophageal cells obtained from Ospedale Policlinico San Martino (Genova, Italia) [56] were cultured in RPMI-1640 and Ham’s F-12 (in a ratio of 1:1) supplemented with 2% FBS, 2 mM glutamine, and 1% penicillin–streptomycin and incubated at standard culture conditions (37 °C and 5% CO_2_). To perform mucoadhesion tests, 2.0 × 10^4^ cells were plated on a 96-well plate.

The permeability assay was performed on an in vitro model of human reconstructed esophageal epithelium (HO2E, purchased from EPISKIN, Lione, France) reconstructed after 5 days of airlift culture of the K510 cell line (derived from human esophageal squamous cell carcinoma) on inert polycarbonate filters in a chemically defined medium. On day 5, the tissues’ inserts were placed at room temperature in a multi-well plate filled with a nutrient agarose solution, in which they were embedded for shipment. After arrival, the HO2E tissues were removed from the agarose under a sterile airflow cabin. The inserts were rapidly transferred to a 6-well plate prefilled with specific growth medium and incubated at 37 °C, 5% CO_2_, and 95% relative humidity [57].

### 4.3. Experimental Protocol

The experiments were divided into three steps: the first one investigated the effects of different XPPA dosages on a GTL-16 cell, analyzing cell viability and ROS production in a dose-response study from 1 h to 4 h. In this step, the cells were also plated in the Transwell^®^ system to verify gastric integrity by TEER measurement following the treatments. The same in vitro model was used to analyze TJ activity, specifically Claudin-1, occludin, and ZO-1, through ELISA at 4 h [58]. The cells were treated with HCl (pH 3.3) for 30 min to mimic the hyperacidity conditions of GERD, and they were then treated with XPPA (Devintec SAGL, Lugano, Svizzera) at different concentrations for a timeframe from 1 h to 4 h.

In the second phase, the mucoadhesive properties of XPPA were investigated on AGS gastric cells and KYSE-30 esophageal cells. The concentration of 1 mg/mL was chosen after conducting a cell viability test. The mucoadhesion of XPPA was evaluated based on the percentage of lectin-bound glycoprotein inhibition. For this purpose, a biotinylated lectin (concanavalin A, Merck Life Science, Rome, Italy), a protein found in some legumes (*Canavalia ensiformis*) that has a high affinity for mannoside and glucoside residues of membrane glycoproteins, was used. The binding sites were then detected using streptavidin peroxidase, which, due to its high affinity for biotin, forms a protein-glucosylectin-biotin-streptavidin-peroxidase complex. The complex was then quantified by measuring the color intensity produced by the reaction of o-phenylenediamine oxidation by the streptavidin peroxidase (Merck Life Science, Rome, Italy) [59].

Finally, a study on the protective properties of XPPA on epithelial paracellular permeability was performed on an in vitro model of HO2E with the K510 cell line. HO2E tissues were prewetted with 15 μL of saline for 15 min at room temperature to recapitulate the epithelium conditions and to achieve a homogeneous distribution of the tested device [60]. To replicate GERD damage, the tissues were then stimulated with HCl (pH = 3.3) for 15, 60, 120, and 240 min after being treated with XPPA for 15 min. In the reference control, tissues were left untreated and exposed to HCl. Following each contact period, the tissues were cleaned in phosphate buffer, and the LY assay (Merck Life Science, Rome, Italy) was used to measure the paracellular permeability. Every procedure was carried out in triplicate.

### 4.4. Cell Viability Through MTT Test

Cell viability based on the In Vitro Toxicology Assay Kit (Merck Life Science, Rome, Italy) was assessed at the end of each treatment, as previously described [61]. The absorbance of each solubilized sample was evaluated at 570 nm with correction at 650 nm measured by a spectrometer (Infinite 200 Pro MPlex, Tecan, Männedorf, Switzerland). The results were expressed by comparing them to the control sample (untreated samples were defined as the 0% line) and reported as the means of five independent experiments performed in triplicate.

### 4.5. ROS Production and Measurement

The rate of superoxide anion release was measured using a standard protocol based on reduction of cytochrome C [62] and measuring the absorbance at 550 nm through the spectrometer (Infinite 200 Pro MPlex, Tecan, Männedorf, Switzerland). O_2_ ratio was expressed as the mean ± SD (%) of nanomoles per reduced cytochrome C per microgram of protein compared to the control (untreated samples) of five independent experiments performed in triplicate.

### 4.6. TEER Measurement for Barrier Integrity

TEER is a quantitative measure describing the integrity of the barrier and the electrical, ohmic resistance of the cell layer. Here, GTL-16 cells were seeded on the apical side of a 24-well Transwell^®^ permeable support for 7 days. TEER was continuously measured using an EVOM3 Voltohmmeter (World Precision Instruments, Sarasota, FL, USA), and experiments began when TEER reached ≥150 Ω·cm^2^ [58]. In particular, to mimic GERD conditions, after 7 days, the medium was changed on both the apical and basolateral sides, adding HCl (Merck Life Science, Italy) to the medium to obtain pH 3.3 at the apical side for 60 min, as reported in the literature [63]. Further stimulations were performed in the same manner and under the same conditions, as previously described.

### 4.7. Occludin Quantification Assay

The manufacturer’s instructions for using the human occludin (OCLN) ELISA kit (MyBiosource, San Diego, CA, USA) were followed [64]. After HCl-induced damage, cells were treated with XPPA for 4 h. After lysing GTL-16 cells in cold Phosphate-Buffered Saline (PBS, Merck Life Science, Rome, Italy) 1×, each sample was subjected to a 10 min centrifugation at 1500× *g* at 4 °C, and 100 μL was analyzed. A spectrometer (Infinite 200 Pro MPlex, Tecan, Männedorf, Switzerland) was used to analyze the enzymatic reaction at 450 nm. The data were compared to the standard curve, which spans 0 to 1500 pg/mL. Results were then expressed as a percentage (%) against the controls (0 line) of five separate experiments carried out in triplicate.

### 4.8. Claudin-1 Quantification Assay

A Cusabio Technology LLC (Huston, Houston, TX, USA) ELISA kit was used to measure the amount of human claudin-1 in GTL lysates following the manufacturer’s instructions [65]. After HCl-induced damage, cells were treated with XPPA for 4 h. After lysing GLT-16 cells in cold PBS (Merck Life Science, Rome, Italy) at a ratio of 1×, the samples were centrifuged at 1500× *g* for 10 min at 4 °C. A spectrometer (Infinite 200 Pro MPlex, Tecan, Männedorf, Switzerland) examined and read 100 μL of each sample. Five independent experiments were conducted in triplicate, and the results were expressed as a mean ± SD (%) vs. the control (0 line) after comparing the data to the standard curve, which spans from 0 to 1000 pg/mL.

### 4.9. ZO-1 Quantification Assay

The manufacturer’s instructions for the human tight junction protein 1 (TJP1) ELISA kit (MyBiosource, San Diego, CA, USA) were followed [66]. After HCl-induced damage, cells were treated with XPPA for 4 h. A volume of 100 μL was evaluated for each sample after GTL-16 cells were lysed with cold PBS (Merck Life Science, Rome, Italy) 1× and centrifuged at 5000× *g* for 5 min at 4 °C. A Tecan Infinite 200 Pro MPlex spectrometer located in Männedorf, Switzerland, read the plates at 450 nm. The standard curve, which spans from 0 to 1000 pg/mL, was compared with the collected data, and the outcomes were reported as the means ± SD (%) of five separate tests conducted in triplicate, as opposed to the control (0 line).

### 4.10. Vitronectin ELISA Kit

The Human VTN (Vitronectin) ELISA Kit (FineTest, Wuhan, China) was used to quantify vitronectin following the manufacturer’s instructions on GTL-16 cell lysates. In summary, each well received 100 µL of material, and the plate was incubated for 90 min at 37 °C. Following the conclusion of the incubation period, the material in each well was removed, and wash buffer was used twice to wash the wells. The aforementioned wells were filled with 100 µL of biotin-labelled antibody working solution, and the plate was incubated for 60 min at 37 °C. After the incubation period, the solution in each well was withdrawn, and wash buffer was added three times to wash the wells. After adding 100 µL of SABC working solution to each well, the plate was incubated for 30 min at 37 °C. At the end of five rounds of washing, 90 µL of TMB substrate was added to each well. A plate reader (Infinite 200 Pro MPlex, Tecan, Männedorf, Switzerland) was used to read the plate at 450 nm as soon as 10–20 min had passed after adding 50 µL of stop solution to each well. A standard curve was plotted relating the intensity of the color (OD) to the concentration of standards (ranging from 9.375 to 600 pg/mL), and the results were expressed as means ± SD (%) versus controls (0 line) of five independent experiments performed in triplicate.

### 4.11. Fibronectin ELISA Kit

Fibronectin quantification was determined using the Human FN1 (Fibronectin) ELISA Kit (FineTest, Wuhan, China) according to the manufacturer’s instructions on GTL-16 cell lysates [67]. A plate reader (Infinite 200 Pro MPlex, Tecan, Männedorf, Switzerland) was used to read the plate instantly at 450 nm. The results were expressed as means ± SD (%) versus controls (0 line) of five independent experiments conducted in duplicate. A standard curve was drawn relating the intensity of the color (OD) to the concentration of standards (range from 1.563 to 100 pg/mL).

### 4.12. Mucoadhesion Test

A product’s mucoadhesion to a specific mucosa can be assessed by calculating the percentage of lectin-binding glycoprotein inhibition. The binding sites were identified using streptavidin peroxidase, which forms a complex protein-glucosylectin-biotin-streptavidin-peroxidase due to its strong affinity for biotin. The following reaction, which results from the oxidation of o-phenylenediamine, quantifies the complex because peroxidase is present:

$$o-phenylenediamine\begin{array}{c} H_{2}O_{2}\\ \underset{Streptavidin\;peroxidase}{\to } \end{array}2,3-diaminophenazine(yellow)$$

Cells were pre-treated with XPPA (1 mg/mL) for 30 min. The concentration was selected based on the results of the initial cytotoxicity test (not shown). A series of cells were treated with a material known to have mucoadhesive activity (PC, positive control, 1% hyaluronic acid). In contrast, another cell series was maintained in a culture medium and received no treatment (NC, negative control). Every experiment was carried out three times. Following the conclusion of the treatment period, the cells received Concanavalin A, streptavidin peroxidase, and o-phenylenediamine + H_2_O_2_ in order. In addition, the cells underwent multiple PBS washes in between each dose. The optical density was measured at 450 nm. The ability of the sample to prevent concanavalin A from binding to the glycosidic residues in the cell membranes is what determines its mucoadhesive; as a result, it is inversely proportional to the optical density that is measured at the end of the reaction and can be computed using the following formula:

$$Mucoadhesivity\left(\%\;of\;NC\right)=\left[\left(1-\frac{{OD}_{sample}}{{OD}_{NC}}\right)*100\right]*100$$

### 4.13. Lucifer Yellow Assay

The paracellular permeability of cells can be studied using LY, a fluorescent dye impermeable to cell membranes that can pass through tissues exclusively via the paracellular route. The flow of LY is substantially higher when TJs are damaged and/or loose, while it has a very poor permeability when the physiological epithelial integrity is preserved. Therefore, 500 μL of LY (500 μM in saline) was administered on the apical side of a human reconstructed esophageal epithelium, and the basolateral compartment was filled with 1 mL of saline solution. Spectrophotometric analysis was used to quantify the amount of LY that had moved from the apical to the basolateral compartment during an hour of incubation at 37 °C [68]. The following formula was used to determine the LY flux:

$$LY\;fux\left(\%\right)=\frac{{RFU}_{BL}}{{RFU}_{APt=0}}\times 100$$

RFU_BL_: fluorescence in the basolateral compartment

RFU_APt = 0_: fluorescence of LY solution applied in the apical compartment

### 4.14. Statistical Analysis

One-way analysis of variance (ANOVA) and Bonferroni post hoc tests were used to process the data acquired using Prism GraphPad statistical software 9.4.1. A Student’s *t*-test with two tails was adopted to compare the two groups. A two-way ANOVA was conducted to evaluate multiple group comparisons, followed by a two-sided Dunnet post hoc test. The means ± SD of at least five independent variables were used to express all results.

## 5. Conclusions

Protecting and restoring a functional esophageal epithelial barrier might be a promising therapeutic approach for GERD patients. The findings of the present work demonstrate the ability of XPPA to form a mucoadhesive and mucoprotective mechanical barrier that promotes TJ integrity and restores esophageal permeability after induction of GERD-like damage. This suggests that XPPA could represent an alternative sustained protective treatment for those patients suffering from GERD, able to promote the strengthening of the esophageal physiological barrier.

## Data Availability

The original contributions presented in the study are included in the article; further inquiries can be directed to the corresponding author.
